# Experimental Researches on the Action of Quinine, Especially in Large Doses

**Published:** 1850-02

**Authors:** 


### MATERIA MEDICA AND THERAPEUTICS.

*Experimental Researches on the action of Quinine, especially in large doses. A Memoir submitted to the Academy of Science.* By M. Brecquet. Report of MM. Andral, Rayer, and Lallemand.—M. Brecquet records the effects on the principal organs of the animal economy, of sulphate of quinine, in doses of fifteen grains and upwards. His experiments have been made upon living animals; to these he adds observations on patients to whom he has administered the remedy in the above-named doses.

1. *Effects on the organs of circulation.*—These were of two kinds,—first, as regards the frequency; secondly, as regarded the force of the pulsations of the heart. The frequency of the pulse was variously reduced from eight to forty beats in the minute.

Alterations in the force of the heart's action were observed by the aid of M. Poisseuille's hæmadynamometer applied to the carotid artery of animals, in whom at the same time solution of sulphate of quinine was injected into the left jugular vein. Varying with the quantity injected, the force was observed to be diminished from a seventh to a tenth, a fourth, a third, and a half; and at last, on injecting thirty grains of the bisulphate in about four ounces of water, all pressure disappeared, the heart's action ceased, and instant death by syncope ensued. These effects were observed to follow regularly, whether the quinine were administered by injection into the vessels, by the stomach, or by insertion into the cellular tissue.

2. *On the nervous centres.*—Injected directly towards the brain by the carotids or ascending aorta, great cerebral excitement and convulsions were produced. If the quinine reached the brain by the more indirect route of the general circulation, agitation, headache, vertigo, tinnitus aurium, paralysis of the nerves of the special senses, muscular twitching and subsultus tendinum, apparent intoxication, then general collapse and loss of all voluntary power. Dissection generally disclosed great congestion of the brain and its membranes, and even meningitis.

3. *On the organs of respiration.*—No appreciable effect was observed, except what might be referable to the slower propulsion of the blood by the heart.

4. *On the digestive organs.*—Inflammation of the mucous membrane, attended with its usual symptoms, though not generally of a severe character.

5. *On the urinary apparatus.*—Pain, frequent micturition, hæmaturia, dysuria, and retention, have been noticed, but always in a slight degree.

6. *On the organs of generation.*—Uterine hæmorrhage of the female, and debility of the organs in the male.

7. *On the skin and the subcutaneous cellular tissue.*—Numbness and coldness of the surface, ecchymosis and petechiæ, more or less extensive.

8. *On the blood and other animal fluids.*—When blood drawn from the vessels was placed in contact with solution of quinine, it became liquefied, and the globules were destroyed; but in order that such effects should take place in the living body, the presence of a much greater quantity than can be taken by the stomach would be required. Animals poisoned by this medicine did not present this liquid state of the blood, but an increase in the proportion of fibrin was found. No trace of quinine could be discovered in the milk or mucous secretions.

The absorption and elimination of quinine in reference to its therapeutical employment, was ascertained by noting the period at which a precipitate appeared in the urine, on the addition of the bi-iodide of potassium, and by observation of the symptoms referable to the nervous system. Thus it was observed that the sulphate in doses exceeding three grains is absorbed in from half an hour to two hours, and produces its physiological effects in another hour. These will continue for about half an hour. A dose of fifteen grains in six hours continues to manifest its influence for from five to six hours. Thirty grains administered in two hours, produce symptoms lasting from twelve to fifteen hours. When the sulphate has been administered for several days, the effects continue many days after it has been withdrawn. The medicine is completely eliminated at the end of ten or twelve hours after small doses, and in about forty-eight or seventy-two hours after large doses.

Women and children are more susceptible of its influence than men; and the stature and strength of the individual modifies its effects. Loss of blood increases also its influence, diminishing its stimulating, and increasing its depressing action. Opiates act in a similar manner, while alcoholic stimulants have a reverse operation.

In reference to its therapeutic properties, M. Brecquet found that the sulphate is the most active of all preparations of quinine; that the alkaloid itself has an action identical with the sulphate, as has also cinchonine, but that the latter is by one-third less powerful; that quinoidine has the same action as quinine on the nervous system, but is much more irritating to the alimentary canal.

M. Brecquet found the solution of sulphate more active by one-half than the same compound in the dry state. Administered by enemata, absorption was found to take place more rapidly than when it was given by the mouth, but the effects lasted a shorter time, and the alkaloid scarcely produced its physiological action. Employed for frictions, ointments, lotions, and other endermic methods, the absorption was very feeble, and no physiological action whatever could be traced.

The physiological and therapeutical effects of this medicine were more regularly and powerfully obtained by its administration in repeated doses; its exhibition therefore requires to be continued for a certain period.—*Lond. Med. Gaz., from Comptes Rendus.*

